# Recent progress in human telomerase structure and its therapeutic targeting

**DOI:** 10.3389/fmolb.2025.1681988

**Published:** 2025-12-04

**Authors:** Muaath Ebrahim Almansoori, Abdulrahman Awad, Sarah Dhaiban, Mohammed Turki Alduhoori, Humayun Sharif, Abdulrahim Sajini

**Affiliations:** 1 Department of Biological Sciences, Khalifa University of Science and Technology, Abu Dhabi, United Arab Emirates; 2 Department of Biomedical Engineering & Biotechnology, Khalifa University of Science and Technology, Abu Dhabi, United Arab Emirates; 3 Department of Biology, Chemistry & Environmental Sciences, American University of Sharjah, Sharjah, United Arab Emirates

**Keywords:** telomerase, imetelstat, telomere, cancer, structural biology, cryo-electron microscopy, drug discovery

## Abstract

Most cancer and stem cells activate telomerase to preserve critical genetic material during cell division. Telomerase is a reverse transcriptase ribonucleoprotein that adds telomeric repeats to chromosome ends, thus overcoming the end-replication problem. Shortening of telomeric repeats, or telomeres, is associated with genomic instability, cancer, and aging. Telomerase dysfunction during early development leads to telomeropathies such as dyskeratosis congenita, pulmonary fibrosis, and aplastic anaemia. Recent advancements in cryo-electron microscopy and improved strategies for purifying human telomerase have laid a strong foundation in the structural biology of telomerase, advancing our understanding of its molecular interactome. In this report, we review the latest progress in human telomerase structure and outline emerging therapeutic strategies targeting telomerase.

## Introduction

It is estimated that 20% of human adults will reach the age of 65 by 2050 (Dogra et al., 2022). Aging reduces the quality of social wellbeing, cognitive function, sleep, and physical activity, thereby making a normal daily routine a burden (Dogra et al., 2022). The process of aging is directly caused by cellular senescence due to telomeric DNA shortening that induces continuous DNA damage response (Rossiello et al., 2022). Telomeric DNA, or telomeres, shorten as the replication of linear DNA is incomplete (Ohki et al., 2001; Yılmaz and Bozkurt, 2024). Also, telomerase, which is responsible for telomere addition, becomes inactive almost in all somatic and mature cells, especially after a time point during development (Ohki et al., 2001; Yılmaz and Bozkurt, 2024; Ulaner and Giudice, 1997). However, some cells retain telomerase activity as they need to divide more frequently than other cells, including stem or progenitor cells, lymphocytes, adult testes and ovaries, and human hair follicle cells (Wright et al., 1996; Hiyama et al., 1995; Ramirez et al., 1997; Morrison et al., 1996). Telomerase and telomeres, on the other hand, provide cancer cells with indefinite replication power (immortalization) (Low and Tergaonkar, 2013). Understanding the biology of telomerase and telomeres is essential for developing strategies for healthy aging and cancer-based therapies.

Telomerase is a ribonucleoprotein (RNP) that binds to chromosome ends and adds telomeres, a tandem repeat of G-rich nucleotides (dTTAGGG) (He and Feigon, 2022). The telomeric DNA is coated with 6 different protein subunits called the shelterin (Yılmaz and Bozkurt, 2024; Welfer and Freudenthal, 2023). This complex helps in the formation of a t-loop, a lariat-like structure that protects DNA and prevents DNA damage responses (Welfer and Freudenthal, 2023; Tomaska et al., 2019; Loukopoulou et al., 2024). DNA damage can initiate when telomere lengths are shortened to critical lengths leading normal cells to senescence or pre-cancer cells to die (Rossiello et al., 2022; Jones-Weinert et al., 2024). Cancer cells overcome this machinery and escape cell senescence by overexpressing telomerase or by alternative lengthening of telomeres (ALT) (Shay, 2016). Mutations in telomerase components, or dysregulated telomerase activity, are associated with aging and cancer (He and Feigon, 2022). Recent high-resolution structures have further delineated telomerase mechanisms, the effects of disease-associated mutations, and specific functions of the human telomerase holoenzyme (Nguyen, 2021; Ghanim et al., 2021; Ghanim et al., 2024). In addition, high resolution of telomerase complex structures can help to understand the mechanism action of telomerase-targeted drugs (Liu B. et al., 2022). In this review, we layout the advances in human telomerase structures, functions, and delve into the role of telomerase structure in drug discovery process. We also shed light on telomerase-targeted drugs, highlighting imetelstat, the only telomerase inhibitor approved by the FDA (Platzbecker et al., 2024).

## Recent understanding of the human telomerase complex structure

**FIGURE 1:**
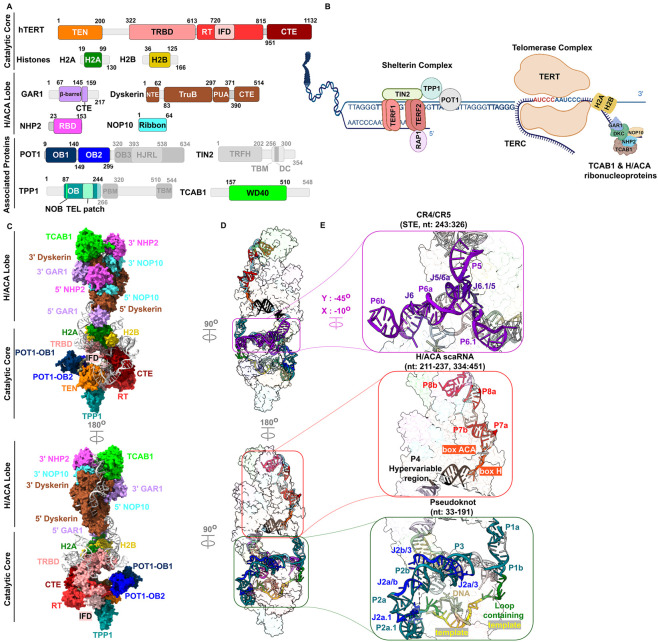
Overall structure of the human telomerase. **(A)** Domain schematics of the catalytic core, H/ACA lobe, and telomerase (TCAB1) or telomere-associated proteins (TIN2-POT1-TPP1). Greyed out portions (e.g., TIN2) indicate unresolved part or protein. **(B)** Simplistic representation of the shelterin and telomerase complexes at the chromosome ends. The other set of H/ACA proteins (GAR1, dyskerin, NOP10, NHP2) are not shown. **(C)** The structure of the telomerase holoenzyme. TPP1 and POT1 are interacting partners with the catalytic core but are not part of it. **(D)** Side views of the holoenzyme where the DNA and hTERC are shown. **(E)** hTERC domains and constituents are highlighted. Catalytic core PDB: 7QXB, H/ACA lobe: 8OUE. human Telomerase Reverse Transcriptase (hTERT), human Telomerase RNA Component (hTERC), N-terminal domain (TEN), telomerase RNA-binding domain (TRBD), Reverse transcriptase (RT), and C-terminal extension (CTE), Insertion in finger domains (IFD), Oligonucleotide binding domains 1, 2, and 3 (OB1, OB2, OB3), Holiday junction like resolvase (HJRL), Telomeric repeat factors homology (TRFH), TRFH-binding motif (TBM, in TIN2), Dyskeratosis Congenita hotspot (DC), N terminus of OB domain (NOB), TPP1 glutamate and leucine rich (TEL) patch, POT1-binding motif (PBM), TIN2-binding motif (TBM, in TPP1), tRNA pseudo-uridine synthase B-like (TruB), Pseudo-uridine synthase and archaeosine transglycosylase (PUA).

The human telomerase complex is composed of a catalytic core and H/ACA lobe. It is also regulated by telomerase and telomere-associated proteins (Figure 1A). The catalytic core comprises the human Telomerase Reverse Transcriptase (hTERT), histones, and parts of the human Telomerase RNA Component (hTERC). hTERT has a modular architecture with multiple functional domains including the N-terminal domain (TEN), telomerase RNA-binding domain (TRBD), Reverse transcriptase (RT), and the C-terminal extension (CTE) domain (Figure 1A) (Nguyen, 2021). TEN domain is responsible for telomerase recruitment and repeat-addition processivity (RAP) (Lambert-Lanteigne et al., 2023). RAP is a unique characteristic of telomerase, in contrast to the nucleotide addition processivity (NAP), which is a shared feature with other polymerases (Moriarty et al., 2004). NAP (type I processivity) is the process of successive nucleotide addition, whereas RAP (type II processivity) is defined as the repetitive copying of template RNA to form multiple telomeric repeats on a single substrate (Moriarty et al., 2004). The hTERT binds to hTERC through the TRBD domain (Nguyen, 2021). The RT domain is the main site of telomere formation and catalysis (Nguyen, 2021). TERT contains a unique domain, called the insertion in the fingers domain (IFD) which aids in enzyme activity and recruitment to telomeres (Chu et al., 2016). The CTE domain, also known as the thumb domain, helps in telomere elongation and stabilizes the binding of DNA-RNA hybrid duplex (Hoffman et al., 2017). Lastly, histones H2A-H2B and their potential contribution to the telomerase complex are discussed in the following section.

The H/ACA lobe is made of two identical complexes, each composed of dyskerin, NOP10, GAR1, and NHP2 subunits, distinguished by their location to 3′ or 5′ end of H/ACA RNA (Nguyen, 2021). They mainly stabilize the whole complex, assist in telomere maintenance, as well as being responsible for telomerase biogenesis (Schmidt and Cech, 2015). NOP10 and NHP2 ease hTERC recognition and binding to dyskerin (Nguyen, 2021). Given that, the H/ACA and telomeric proteins are important regulators of telomerase function. However, they are not necessary for enzymatic activity. Indeed, *in vitro* reconstitution experiments showed hTERT and hTERC are sufficient for minimal telomerase activity (Weinrich et al., 1997).

Telomere- or telomerase-associated proteins interact with the catalytic core or H/ACA lobe and play significant roles in telomerase function and stability (Figure 1B). For example, telomerase Cajal body 1 (TCAB1) is a telomerase-associated protein which engages with the H/ACA lobe facilitating telomerase recruitment and its access to Cajal bodies (Stern et al., 2012). Recently, it was found that TCAB1 prevents hTERC accumulation in the nucleolus, keeping and associating the hTERC pool with hTERT, which leads to high activation of the telomerase machinery (Klump et al., 2023). The TCAB1 and H/ACA RNPs are involved in other pathways including P53 regulation and pseudo-uridylation (Sun et al., 2014; Hamma and Ferré-D'Amaré, 2010; Schlotter et al., 2023). On the other hand, telomere-associated proteins, such as the shelterin, pose different binding modes. For example, TRF1 and TRF2 bind to the double stranded telomeric DNA (Nishikawa et al., 2001; Broccoli et al., 1997). In contrast, POT1 recruits TPP1 and binds to the single stranded telomeric DNA (Liu et al., 2004). TIN2 binds to both TRFs as well as to TPP1 (Ye et al., 2004; Hu et al., 2017). RAP1 is recruited to telomeres by TRF2, to which it binds (Li et al., 2000). Collectively, the telomerase holoenzyme and associated proteins assemble at the telomeric DNA sites (Figure 1B), to regulate telomere synthesis cycle (Nguyen, 2021; Wu et al., 2017). The TPP1-POT1 complex mainly interacts with the TEN domain and the 3′ single stranded telomeric DNA to recruit the telomerase (Zinder et al., 2022; Schmutz and De Lange, 2016; Sekne et al., 2022). Additionally, TIN2 stimulates telomerase processivity with TPP1-POT1, and maintains cohesion of the shelterin complex (Hu et al., 2017; Kaur et al., 2021; Pike et al., 2019). These proteins (TIN2, POT1, and TPP1) are the only known shelterin components that stimulate telomerase processivity; therefore, they were reconstituted with telomerase to obtain the most complete holoenzyme model to date (Figure 1C, TIN2 was not solved in the latest structures) (Sekne et al., 2022).

hTERC is a non-coding RNA that tethers both the catalytic core and H/ACA structural units and hence, it has some domains belonging to the catalytic core while the others in the H/ACA lobe. The hTERC secondary structure contains 3 domains: Pseudoknot - CR4/CR5 - H/ACA small Cajal body RNA (scaRNA) domain (Figures 1D,E) (Chen et al., 2000; Zhang et al., 2011; Ivanyi-Nagy et al., 2018). The pseudoknot domain is located in the catalytic core. It holds the template RNA which is reversely transcribed for telomere synthesis (Theimer et al., 2005). Furthermore, the template is surrounded by flanking regions which regulate precise RAP and enzymatic activity by altering pseudoknot structure (Theimer et al., 2005). Indeed, the folding and conformation of pseudoknot domain determines the telomerase activity (Theimer et al., 2005; Chen and Greider, 2005; Mihalusova et al., 2011). CR4/CR5 domain is another site where hTERT anchors to hTERC in the catalytic core (Boyraz et al., 2016). Some studies point out that CR4/CR5 residues are possibly pseudo-uridylated (Kim et al., 2010; Schwartz et al., 2014; Zemora et al., 2016). The H/ACA scaRNA domain located in the H/ACA lobe. It has 4 important motifs, mainly the H box, ACA box, Cajal body (CAB) box, and biogenesis promoting box (BIO box) (Hukezalie and Wong, 2013). The H box and ACA box form a hairpin-hinge-hairpin-tail structure that attracts complete set of H/ACA RNPs binding to each hinge (Schlotter et al., 2023). The CAB box is a designated motif for scaRNAs, in which it allows entry to Cajal bodies (Stern et al., 2012). The BIO box is responsible for hTERC processing and maturation (Ketele et al., 2016).

The current understanding of the telomerase structure arises from building on previous work and improvements in structural biology techniques. This sheds light on several and novel intra-domain and protein-protein interactions within the telomerase. They will be discussed in the following section.

## Timeline of human telomerase structures

**FIGURE 2:**
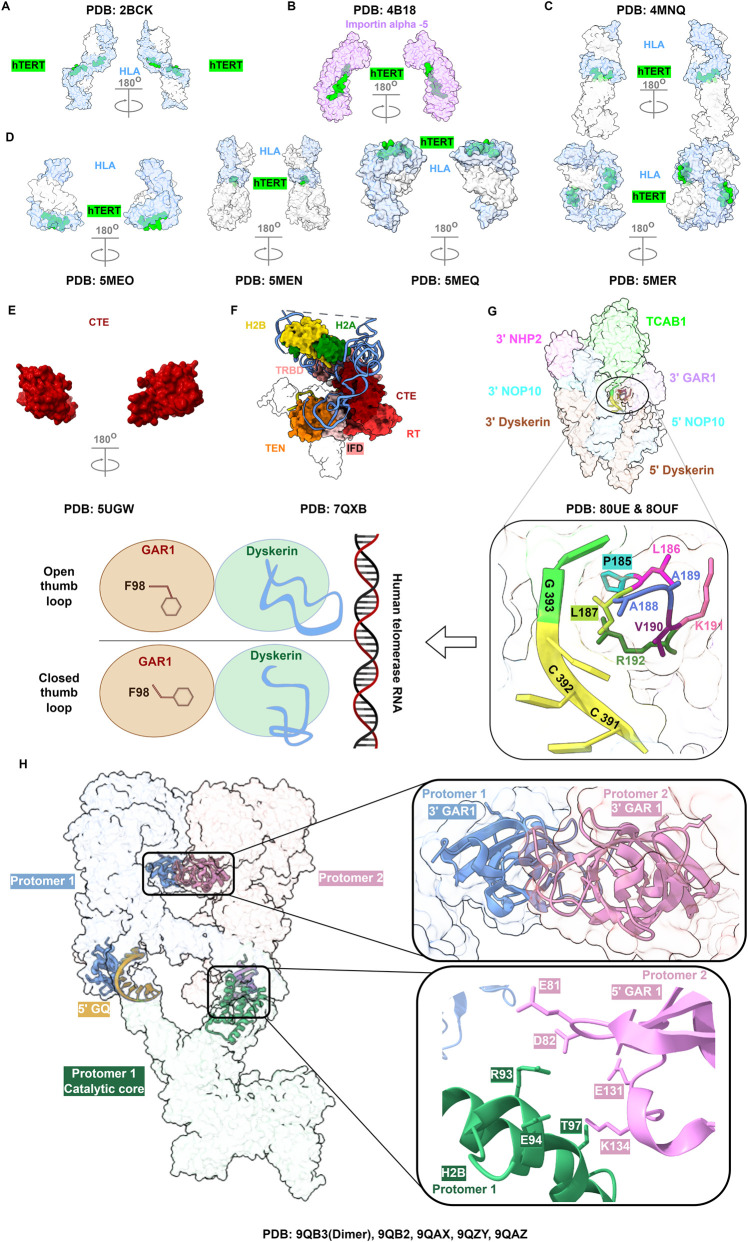
Progression of telomerase 3D structures. **(A)** Telomerase peptide bound to human leukocyte antigen (HLA). **(B)** The Telomerase NLS peptide signal solved with importin-*α*5. **(C)** Telomerase-HLA complex in complex with T cell receptor (TCR). **(D)** Same with CD8 clone TCR recognizing telomerase peptide. Greyed-transparent parts in **(C,D)** correspond to *β*2microglobulin and/or TCRs. **(E)** The C-terminal extension domain (CTE) of hTERT. **(F)** hTERT and histones structure without TPP1-POT1 (greyed out). **(G)** H/ACA lobe, dyskerin thumb loop, and hTERC are highlighted in the closed state. The schematic illustrates the conformational changes in the thumb loop and the F98 of GAR1 in open and closed states. **(H)** Telomerase dimer where full protomer 1 and the H/ACA of protomer 2 are shown. Two interfaces are shown: The first one is between 3′ GAR1 from protomer 1 and protomer 2, and the second is between histone H2B of protomer 1 and the 5′ GAR1 of protomer 2. The location of the 5′ G-quadruplex (GQ) is shown as well.

Given its role in tumorigenesis, peptides derived from hTERT were promising antigens in cancer immunotherapy (Arai et al., 2001; Cole et al., 2006). Hence, peptides were used to solve and study human leukocyte antigens (HLA) (Figure 2A) (Cole et al., 2006). A similar approach showed that importin-*α* proteins interact with hTERT’s bipartite nuclear localization signal (NLS) (Figure 2B) (Jeong et al., 2015). The NLS, amino acids (aa) 222–240, lies within the proline/arginine-rich linker (PAL; 201–325 aa), supporting a functional role for the PAL region in hTERT nuclear transportation (Jeong et al., 2015; Balch et al., 2025). Also, using hTERT peptides in complex with HLA allowed the study of T cell cross reactivity, and interactions at T cell receptor (TCR) with the major histocompatibility complex (Figures 2C,D) (Cole et al., 2014; Cole et al., 2017). By using cryogenic electron microscopy (cryo-EM), Sauerwald et al were able to resolve the whole telomerase holoenzyme structure, albeit at very low resolution (30Å) (Sauerwald et al., 2013). They showed a dimeric bilobal structure indicating that the telomerase is dependent on both subunits. A dimeric model of telomerase (one hTERT and hTERC on both lobes) was supported by their findings in the experiments. Later, Hoffman *et al.* solved the thumb/CTE domain of hTERT (Figure 2E) (Hoffman et al., 2017). They found enlarging the constructs to include the thumb loop and a portion of motif E-I produced insufficient protein yield for X-ray crystallography. Although the poor sequence conservation between human and Tribolium CTEs, Hoffman *et al.* showed that the human CTE is structurally conserved with the thumb domain of Tribolium telomerase (root mean square deviation: 2.5Å). They share similar helical bundle formation with three conserved motifs and conserved hydrophobic pocket (E-I, E-II, E-III, and FVYL pocket) (Hoffman et al., 2017).

After the advancements in telomerase purification strategies, and detection techniques [resolution revolution (Kühlbrandt, 2014)] in Cryo-EM, Nguyen et al. were the first to solve the telomerase holoenzyme with resolutions higher than 10Å (Nguyen et al., 2018). They revealed a monomeric telomerase with a bilobal architecture; the second lobe in their cryo-EM map was assigned to the H/ACA lobe bound to TCAB1 (Nguyen et al., 2018). They demonstrated two H/ACA heterodimers bridged to hTERT by P1a and P4.2 stems of hTERC. Later in 2021, Ghanim *et al.* solved the telomerase holoenzyme with resolutions higher than 4Å (Figure 2F) (Ghanim et al., 2021). All the studies from this time report almost, if not, the same H/ACA lobe (Figure 1C), so hereinafter the H/ACA lobe structure will not be cited (Ghanim et al., 2021; Liu B. et al., 2022; Sekne et al., 2022; Wan et al., 2021). They identified the H2A–H2B histone dimer as a component of the human telomerase holoenzyme, validated by an oligonucleotide immunoprecipitation assay. In the same assay, histones H3–H4 showed only weak, inconclusive binding, and they were not resolved in the cryo-EM map. Further investigation is required. H2A-H2B plays a role in the catalytic core where they bind and stabilize the CR4/CR5 domain of hTERC. Also, H2A-H2B histones possibly assist in telomerase recruitment to telomeres after DNA replication. The authors find this notion appealing owing to the association of TERT to the replication fork in telomeric sites (Margalef et al., 2018). Also, the fact that H2A-H2B dimers are deposited after the addition of H3-H4 tetramers in the replicated DNA makes it more appealing (Almouzni et al., 1990). They also considered the possibility of it aiding in RNP assembly. This idea is tempting when considering residues from 5′GAR1 were found to form contacts with H2B histone in recent structures (Balch et al., 2025). The paper also demonstrated a resolved TCAB1 structure, which binds to the P8 stem loop with the help of NHP2. The NHP2-P8 binding is presumed to ease the binding between H/ACA subunits.

In the same year, Wan et al. solved the telomerase structure and found the same components of the complex (Figure 2F) (Wan et al., 2021). On one side, they demonstrated that 5 nucleotides from the P7b stem and neighbouring nucleotides make a bulge that blocks the pseudo-uridylation pocket of 3′dyskerin. The bulge possibly eases recognition of P8 stem to NHP2 and TCAB1. On the other side, and similarly, another 5 nucleotides in the middle of P4 stem blocks the 5′dyskerin pseudo-uridylation pocket. This pushes the 5′H/ACA side towards the catalytic core. The main finding Wan *et al.* demonstrated is the zipper head mechanism by L980. The isobutyl side chain of L980 sits in proximity to the template RNA-DNA duplex (Wan et al., 2021). It disturbs RNA-DNA H-bonding during elongation, thus enhancing RAP and slowing NAP activity. Interestingly, it was shown three base pairs between the DNA substrate and the RNA template are sufficient for catalysis, and the active site accommodates up to seven nucleotides of DNA. This agrees with yeast telomerase data (Förstemann and Lingner, 2005), suggesting a conserved mechanism that constrains the length of the nucleotides within the active site.

In the following year, Sekne et al. solved TPP1, and TPP1-POT1, members of the shelterin complex, with hTERT (Figure 1C, catalytic core) (Sekne et al., 2022). The TPP1 N-terminus of the oligonucleotide/oligosaccharide-Binding (OB) domain (NOB), and the glutamate-leucine rich region, TEL, were shown to be crucial for telomerase recruitment (Sekne et al., 2022). Indeed, they were shown to contribute to telomerase recruitment, RAP, and telomere lengthening (Grill et al., 2018). Both NOB and TEL patch interact with helix-*α*5 of the TEN domain, which localizes in the N-terminal dissociates-of-activities domain of telomerase (N-DAT) (Sekne et al., 2022; Armbruster et al., 2001). Mutations in N-DAT are linked to impairment in telomerase recruitment which results in telomere shortening (Sekne et al., 2022; Armbruster et al., 2001; Sexton et al., 2012). Telomerase recruitment is affected by POT1, more than TPP1, as indicated by contact area substitution experiments (Sekne et al., 2022; Armbruster et al., 2001). However, TPP1-hTERT stabilized more DNA tandem repeats in the structure resolved by Sekne et al. (2022). Authors also suggest a new DNA path (trajectory) that is guided by hTERT, hTERC and POT1 (Sekne et al., 2022). Specifically, this path reveals a DNA anchor site formed by a specific motif in the TEN domain (PLYQ) and *β*17 strand of IFD. Through mutagenesis, they demonstrated that both the strand and the motif could help in RAP (Sekne et al., 2022). Although a construct of TPP1-POT1-TIN2 was used, only TPP1-POT1 proteins were solved.

Subsequently, Liu et al*.* solved for TPP1-hTERT complex and H/ACA lobes (Figure 1C, without POT1) (Liu B. et al., 2022). In line with Sekne et al. results (Sekne et al., 2022), they pointed out the same hTERT-TPP1 charge interaction at TEN domain K78 and TPP1 TEL patch (Liu B. et al., 2022). K78 is one of the main charge-charge interaction residues that are vital for telomerase recruitment as demonstrated in charge swap experiment (Schmidt et al., 2014). The rest of charge/polar interactions at the sites of TEN-TPP1 and IFD-TPP1 were different in both studies, with Sekne *et al.* having more interactions (Liu B. et al., 2022; Sekne et al., 2022). Besides, Liu et al. proposed K499, E565 and R622 to regulate template RNA stepwise insertion by flipping the nucleotides into the active site (Liu B. et al., 2022). Interestingly, E565 is one of the T motif residues that was predicted to influence the enzymatic activity and RAP (Weinrich et al., 1997; Drosopoulos and Prasad, 2010). Additionally, the study illustrated several hTERC-CTE interactions, which are disrupted in disease-causing mutations and by BIBR1532, a selective telomerase inhibitor (Liu B. et al., 2022; Podlevsky et al., 2007).

A breakthrough in solving the telomerase structure occurred recently in obtaining a resolution higher than 3Å (Figure 1C, H/ACA lobe) (Ghanim et al., 2024). The high resolution in the resolved structure revealed major residues involved in dyskerin domains (e.g., NTE), hTERC, and H/ACA RNPs that were missing from previous structures (Liu B. et al., 2022; Nguyen et al., 2018). To elucidate, the dyskerin NTE domain plays a key role in the interactions between 5′ and 3′ H/ACA heterotetrameric proteins (Ghanim et al., 2024). In addition, the NTE and the dyskeratosis congenita-like domain, serve as one of the hotspots of disease-causing mutations for Dyskeratosis Congenita (DC) (Rodriguez-Centeno et al., 2019; MacNeil et al., 2019). DC is a genetic disorder that arises from germline mutations in telomerase components, such as dyskerin/DKC1, which lead to impaired telomerase biogenesis and function, and hence causing telomere shortening during early development and several abnormalities including premature aging and bone marrow failure syndromes (Tummala et al., 2024; Vulliamy et al., 2008). Mutations at these sites can alter the stability and localization of dyskerin, as well as hTERC’s stability (MacNeil et al., 2019; Brault et al., 2013; Qin et al., 2024). Other dyskerin mutation hotspots are within the pseudo-uridine synthase and achaeosine transglycosylase (PUA) domain (MacNeil et al., 2019; Trahan et al., 2010). Dyskerin’s CTE domain is another hotspot (Qin et al., 2024), yet it remains poorly resolved in recent structures (Ghanim et al., 2021; Ghanim et al., 2024; Liu B. et al., 2022).

Furthermore, a key resolved structure was the thumb loop in 3′dyskerin (Figure 2G) (Ghanim et al., 2024). The thumb loop (182–194 aa) is located near the pseudo-uridylation catalytic site, in which conformational changes in the thumb loop determine the pseudo-uridylation efficacy of dyskerin (Duan et al., 2009; Liang et al., 2009; Garus and Autexier, 2021). The thumb loop adopts two conformations in canonical H/ACA RNP: an open state where it binds to GAR1, and a closed state conformation after binding of the substrate RNA, which leads to a conformational change in the thumb loop that stabilizes the interaction with a substrate RNA (Duan et al., 2009; Garus and Autexier, 2021). In other words, canonical H/ACA RNPs have a guiding RNA that contains the H-box and the ACA-box motifs and binds to substrate RNAs for further processing (Hamma and Ferré-D'Amaré, 2010; Caton et al., 2018). Although no known RNA substrate for telomerase, Ghanim et al. suggested that the resolved 3′ dyskerin thumb loop corresponds to a semi-closed conformation (Ghanim et al., 2021). In semi-closed state, the G393 moves toward the active site, away from the RNA duplex, resembling the archaeal substrate-bound H/ACA RNP (Ghanim et al., 2024). The study also suggests that a sequence from the P7 stem loop (C431-C436) acts as a 3′ guiding RNA sequence after analogy with the archaeal H/ACA RNP, which differs from Wan et al. who assumed it a 3′pseudosubstrate (Ghanim et al., 2024; Wan et al., 2021) An open conformation of the 3′ thumb loop was also resolved by Ghanim et al., and it has been proposed to have restricted mobility due to the influence of 3′GAR1 (Ghanim et al., 2024). The switch between open and closed thumb loops comes with a re-orientation of the hydrophobic surface due to F98 movement (Figure 2G). Moreover, the high resolution revealed insights about TCAB1 and its interactions with NHP2 (Ghanim et al., 2024), and added more to previous articles (Ghanim et al., 2021; Nguyen et al., 2018). Ghanim *et al.* solved a *β*-hairpin loop (317–326 aa) which contacts and stabilizes P8 stem loop (Ghanim et al., 2024). They also found a loop (483–489 aa) in TCAB1 that interacts with 3′NHP2, hence the name was given as NHP2-interacting loop (NIL) (Ghanim et al., 2024).

While the previous work, except for Saurelward et al., and discoveries were on having a monomeric telomerase holoenzyme (Sauerwald et al., 2013), the latest finding by Balch *et al.* found the telomerase holoenzyme to dimerize (Balch et al., 2025). The dimerization possibly aids in the telomerase assembly. This is supported by the reduced levels of dyskerin when key dimerizing elements, such as P4.2 stem, are mutated. Indeed, the 5′dyskerin from one protomer interacts with hTERC of the other protomer, specifically the junction between P4.2 and P5 stems. Interestingly, mutant P4.2 stem hTERC was still able to form monomeric telomerase, yet these monomeric telomerases had reduced catalytic activity. Mutations in residues contributing to dimerization, like NOP10 (R38W) and Dyskerin (R158W), have been linked to DC (Balch et al., 2025; Walne et al., 2007; Knight et al., 2001).

Besides, the authors showed new insights about GAR1 binding modes in dimerized holoenzymes (Figure 2H). The N-terminal of 3′GAR1 arginine-glycine (RG/RGG) regions of both protomers were forming contacts with P7b stems. RG regions have several features [reviewed elsewhere (Chong et al., 2018; Thandapani et al., 2013)], in which RNA binding is one of the prominent ones (Kiledjian and Dreyfuss, 1992). In addition, 5′GAR1 makes contacts with H2B histone in the catalytic core, possibly forming charge-charge interactions that might be aiding in telomerase assembly. The 5′GAR1 also stabilizes 5′hTERC first nucleotides (5′ leader sequence, 1–31 nucleotides) of hTERC which shape P1 stem and G-quadruplex structure (Balch et al., 2025; Gros et al., 2008). Wan et al. however, visualized them as a stem (Wan et al., 2021). This could suggest alternative forms of hTERC folding might determine telomerase holoenzyme state. Previous studies suggest that G-quadruplex is formed in hTERC precursor, and it is recognized by RHAU/DHX36 (G-quadruplex helicase) to increase hTERC accumulation and P1 helix formation (Gros et al., 2008; Booy et al., 2012; Lattmann et al., 2011; Sexton and Collins, 2011). Nonetheless, the work of Balch et al. suggests independence of hTERT activity from dimerization. Collectively, it is plausible to say that human telomerase dimerization is an intermediate assembly product that requires further processing to produce an active monomeric telomerase holoenzyme. However, there is some evidence suggesting mature and active telomerase is a dimer where the catalytic activity is dependent on both subunits (Sauerwald et al., 2013; Wenz et al., 2001; Cohen et al., 2007; Arai et al., 2002).

**FIGURE 3:**
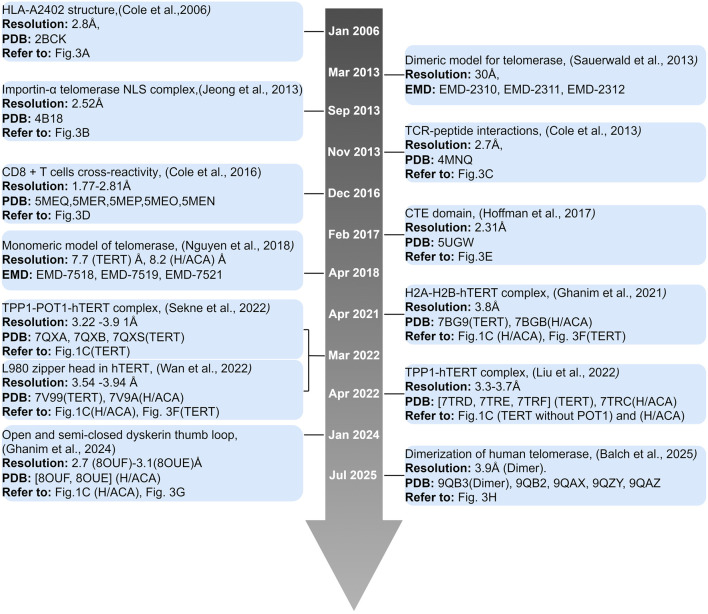
Timeline of human telomerase structures. Timeline of telomerase structure from January 2006 to July 2025. Main finding is indicated with reference, resolution in Å, PDB or EMD ID, and reference in Figures 1, 2 if present.

Resolving the telomerase structure went through multiple stages (summarized in Figure 3). There is still a need for enormous work to identify and solve for a full intact telomerase complex model to understand the RNP dynamics. Novel and effective strategies in telomerase regulation will emerge in cancer treatment, but only if we understand the interactions governing the whole telomerase complex.

## Telomerase and related protein’s structure and drug discovery process

The merits of having high-quality 3D structures are beyond understanding the science of the macromolecule. They offer a broad translational benefit for drug development, discovery and binders’ design. For example, the co-crystal structure of KRAS, a key player in pancreatic tumorigenesis (Liu Y. et al., 2022), with binders enabled the identification of new pockets and the emergence of the first FDA-approved drugs against KRAS (G12C): sotorasib (AMG 510) and Adagrasib (MRTX849) (Canon et al., 2019; Gentile et al., 2017; Fell et al., 2020).

**FIGURE 4:**
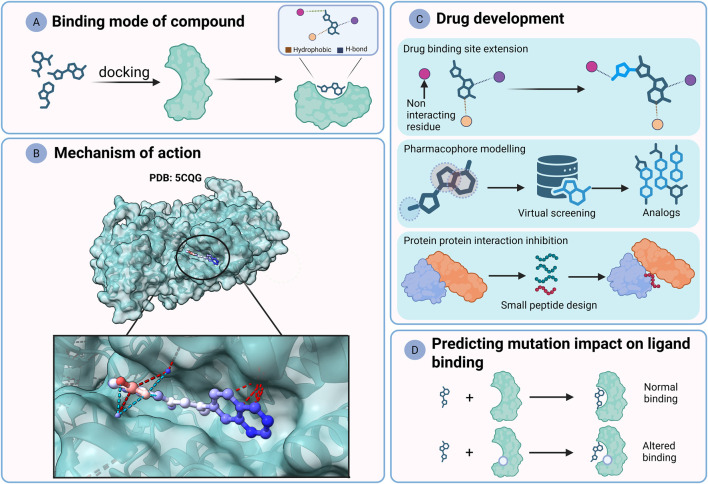
The roles of telomerase and related proteins 3D structures in telomerase-targeted drug development. The existence of telomerase structure aided the advancement of drugs targeting the telomerase. **(A)** Virtual docking of chemical compounds into the binding site can reveal the binding mode which shows the chemical interactions between the compound and the amino acids. **(B)** The co-crystal structures of the highly selective telomerase inhibitor (BIBR1532) can reveal their mechanism of action with *Tribolium Castaneum* telomerase. **(C)** Upper panel: Docking and 3D visualization of the pocket could guide for deriving new chemical moieties that extend the compound binding range to new residues. Middle panel: Pharmacophore spheres predict the interactive chemical moieties with the receptor within the compound. Then, virtual screening occurs in virtual libraries for compounds with similar pharmacophores. The compounds are modified based on the findings. Lower panel: Protein-protein interactions can be analysed *in silico* to design small peptides that target the complex. **(D)** In silico mutagenesis of telomerase structure can predict the impact of the mutation on ligand binding.

Regarding telomerase, many studies have tried to investigate several compounds that potentially possess anti-cancer activity, such as dihydropyrazole, pyrazole–pyrimidine, and anthraquinone derivatives (Gillis et al., 2008; Maciejewska et al., 2022; Wu et al., 2014). After finding the best set of inhibitors, they are docked to the telomerase to check the compounds binding mode (Figure 4A). The binding mode shows the compound orientation and the corresponding interaction between the binding site and the compound. As the human telomerase 3D structure was not resolved or have just started to emerge, some studies used Tribolium catalytic subunit (PDB: 3DU6) for investigation (Gillis et al., 2008). Others utilized a human telomerase (TERT) homology model (Steczkiewicz et al., 2011) to understand the binding of the compounds (Wang et al., 2016; Xue et al., 2015). Homology modelling predicts protein 3D structure based on known protein structures from homologous proteins. One study utilized homology modelling to build their human dyskerin model (Armando et al., 2018). They achieved around 40% telomerase inhibition by targeting a pocket containing the K314 residue. This residue is mutated during DC and leads to telomerase inhibition (Zeng et al., 2012). Docking alone is prone to generating inaccurate results (Berry et al., 2015). To have a better understanding of telomerase inhibition by small compounds, efforts have been made to elucidate the mechanism of BIBR1532, a highly selective telomerase inhibitor (Damm et al., 2001; Bryan et al., 2015). Although the good selectivity of the BIBR1532 against the telomerase, it requires improvements to enhance its pharmacokinetics to be considered for the clinical trials (Barma et al., 2003). Co-crystallization of BIBR1532 with the Tribolium telomerase (PDB: 5CQG) provided the binding site and the optimal binding mode between the compound and the site residues (Figure 4B) (Bryan et al., 2015). Now we understand that the BIBR1532 binds to a conserved hydrophobic pocket, namely, the FVYL through hydrophobic interactions. Disrupting the pocket residues leads to telomere shortening and reduced telomerase activity. This was attributed to possible disruption in telomerase RNA component (CR4/CR5 domain) binding to the pocket. Similar efforts have been made to find nucleoside analogs to inhibit telomerase activity. This resulted in another co-crystal structure between 5-MeCITP and the active site of Tribolium active site, revealing new inhibition mechanism of telomere addition (Hernandez-Sanchez et al., 2019).

While both strategies are powerful and useful on their own, they can guide drug development. For example, docking BIBR1532 to Tribolium telomerase motivated Xue et al. to add methoxy group to enhance compound interactions (Figure 4C, upper panel) (Xue et al., 2015). Also, the BIBR1532 co-crystal structure enabled pharmacophoric based designs where algorithms predict the interactive moieties from the bound drug. This can be followed by virtual screening where compounds posing similar pharmacophores are identified and subsequently tested, modified, or used as templates to modify the starting compound (e.g., BIBR1532) (Figure 4C, middle panel). Many papers utilized BIBR1532 co-crystal pharmacophores and structure-based methodologies, and they established new analogs that pose close telomerase inhibition to the BIBR1532 *in vitro* and greater anti-tumour activity than doxorubicin *in vivo* (Liu et al., 2020; Al-Karmalawy et al., 2022; Al-Karmalawy et al., 2023). While the previous papers used animal or human telomerase models, Zuo et al. implemented *in silico* approaches on experimentally resolved human telomerase structure (PDB: 7BGB) (Zuo et al., 2024). They implemented a pharmacophore model in the TCAB1-hTERC interacting residues that ended with a molecule that outperformed the inhibition of telomerase by BIBR1532 (IC50 = 0.03 *µ* M vs. IC50-BIBR1532 = 0.091 *µ*M). Away from the telomerase, targeting telomere-related proteins could regulate telomerase regulation. By *in silico* means, Jaiswal and Lakshmi designed 5 amino acid length small peptide to disturb *Arabidopsis thaliana* POT1 interaction with TRB [similar to human TRF1/TRF2 (Kusov et al., 2023)] (Figure 4C, lower panel) (Jaiswal and Lakshmi, 2015). Docking metrics showed reduced interactions between POT1 and several TRB isoforms (TRB1-3). The results were supported *in silico* mutagenesis study of the interacting residues.

As mentioned previously, germline mutations in the telomerase components could lead to shorter telomeres as what occurs in DC. Kalathiya et al. implemented single point mutations on a human telomerase model and found mutations could either increase or decrease the binding with drugs (Figure 4D) (Kalathiya et al., 2019). This approach could be useful when considering personalized telomerase-targeted therapies, especially for different populations that pose different variants (Lim et al., 2025).

Although many compounds and designs are promising, further experimental follow ups in animal and human models are required to confirm their pharmacokinetics and pharmacodynamics before proceeding with clinical testing and implementation.

## Telomerase inhibitors and their mechanisms of action

**FIGURE 5:**
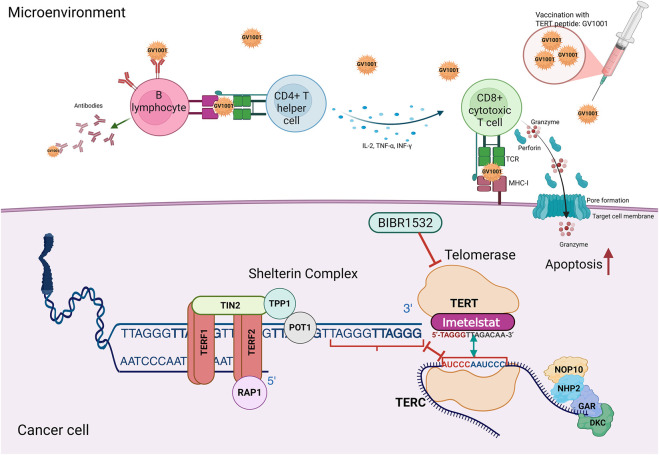
Mechanisms of telomerase Inhibition. Telomerase inhibition can be achieved through immunotherapy and direct enzymatic targeting. Vaccination with TERT peptides, such as GV1001, stimulates an immune response by activating CD8^+^ cytotoxic T cells, which secrete perforin and granzyme to induce apoptosis in cancer cells. These peptides also activate CD4^+^ helper T cells, which enhance CD8^+^ responses through cytokine secretion, while B cells produce antibodies that recognize hTERT, further contributing to immune-mediated telomerase inhibition. Another approach involves direct telomerase inhibition using small-molecule inhibitors like BIBR1532, which block the catalytic activity of telomerase and prevent telomere elongation. Additionally, telomerase activity can also be suppressed by targeting its RNA component (hTERC). Imetelstat, an oligonucleotide, binds to hTERC and competitively inhibits its interaction with the telomeric DNA sequence, preventing the formation of the active enzyme complex required for telomere extension.

The telomerase 3D structures helped in advancing and enhancing telomerase inhibition strategies. However, this was attainable due to the cumulative efforts of previous studies that elucidated the importance of telomerase inhibition and designed the initial set of anti-telomerase drugs. Telomerase detection in human tumours by 1994 emphasized its fundamental role in cancer progression and spurred efforts to develop inhibitors aimed at inducing telomere shortening to limit cancer cell proliferation (Kim et al., 1994). Early studies in the 2000s led to the development of small molecule inhibitors, antisense oligonucleotides, and natural product-based compounds, forming the foundation for more advanced approaches, such as immunotherapies and gene-editing technologies designed to enhance therapeutic precision (Figure 5) (Harley, 2008). Telomerase inhibition results in progressive telomere shortening, ultimately causing senescence or apoptosis in cancer cells (Shammas et al., 2005). This strategy has demonstrated the ability to slow tumour growth and enhance the effectiveness of standard treatments (Shammas et al., 2005). A key advantage of telomerase inhibitors is their selectivity, as normal somatic cells, which have minimal reliance on telomerase, are generally more resilient to telomere shortening compared to cancer cells (Shay and Wright, 2002; Guterres and Villanueva, 2020). Small molecule telomerase inhibitors typically target hTERT or disrupt its interaction with the telomerase hTERC. One of the earliest inhibitors, BIBR1532, binds to the hTERT catalytic site, leading to progressive telomere erosion (Damm et al., 2001). Imetelstat (GRN163L), a lipid-conjugated 13-meroligonucleotide, inhibits the telomerase catalytic activity and prevents telomere elongation (Herbert et al., 2005; Ruden and Puri, 2013). Mechanistically, it acts by competitively binding to the template region of hTERC, thereby inhibiting telomerase enzymatic activity rather than preventing its access to telomeres (Herbert et al., 2005; Ruden and Puri, 2013; Asai et al., 2003). Imetelstat has shown promise in clinical trials, particularly for myelofibrosis and certain solid tumours, although early studies reported adverse effects such as haematological toxicity and hepatotoxicity (Tefferi et al., 2015). In contrast, immunotherapy-based approaches stimulate the immune system to recognize and attack telomerase-expressing cancer cells. The peptide vaccine GV1001 demonstrated potential in phase I and II clinical trials, although its efficacy in larger populations remains inconclusive (Bernhardt et al., 2006). A recent phase III clinical trial have shown combining GV1001 with gemcitabine/capecitabine increased overall survivability and better disease progression in untreated patients characterized with high eotaxin levels and advanced stages of pancreatic cancer (Jo et al., 2024). Another phase III trial showed better urinary symptoms in benign prostatic hyperplasia patients treated with GV1001 than 5 mg finasteride (Shin et al., 2025). Natural compounds, such as alkaloids, have also been identified as telomerase inhibitors. Preclinical studies have found that curcumin can inhibit telomerase, suggesting its potential to expand the range of treatments targeting this enzyme (Khaw et al., 2013). Non-natural nucleosides have shown decent anti-telomerase activity by targeting the active site, resulting in and dysfunctional telomeres (Hernandez-Sanchez et al., 2019). A promising nucleoside is 6-thio-dG, which has been shown to improve anti-cancer effects against Non-Small Cell Lung Cancer (NSCLC) (Eglenen-Polat et al., 2024). Bi-modular molecular design from 6-thio-dG and floxuridine improved immunotherapy against advanced tumors (Mender et al., 2025). Currently, at least two clinical trials are being conducted with 6-thio-dG. The first trial (NCT05208944), initiated in June 2022, is a phase II trial and aims to study the effect of 6-thio-dG administration in association with the PD-1 inhibitor Cemiplimab. The study aims to evaluate whether administering 6-thiodG prior to Cemiplimab can re-sensitize NSCLC cells that are resistant to immunotherapy. A subsequent trial (NCT06908304), scheduled to start in November 2025, aims to confirm the impact of the proposed combination versus chemotherapy, hoping to demonstrate a superior treatment with the 6-thio-dG and Cemiplimab regimen.

Although telomerase inhibitors show promise, they come with several limitations. A significant limitation is the delayed therapeutic response, as telomere shortening must occur over multiple cell divisions before reaching a critical length that triggers cell death (Shay and Wright, 2006). Some cancer cells evade this process by activating the ALT, a telomerase-independent mechanism that sustains continuous proliferation (Shay and Wright, 1996; Bryan et al., 1997; Macha et al., 2022). ALT is particularly prevalent in mesenchymal-origin cancers, such as sarcomas and gliomas, due to their low baseline telomerase activity and higher genomic instability, making them more likely to adopt telomerase-independent mechanisms (Mori et al., 2024; Jiao et al., 2012; Venturini et al., 2010). Molecular and genetic profiling may help identify patient subgroups most likely to benefit from telomerase inhibitors, paving the way for more personalized treatment strategies (Zhang et al., 2020). However, as mentioned earlier, concerns remain about off-target effects and toxicity (Tefferi et al., 2015). Additionally, telomerase plays a crucial role in stem cell maintenance, and its prolonged inhibition may impair tissue regeneration in high-turnover tissues such as bone marrow (Harley, 2008). Critically short telomeres may also lead to genomic instability, potentially giving rise to more aggressive tumour clones (Huang et al., 2024). To address these challenges, combination therapies targeting both telomerase-dependent and ALT pathways, along with molecular profiling to identify patients most likely to benefit, are being explored to improve outcomes (Jafri et al., 2016).

## Imetelstat: an FDA approved telomerase inhibitor drug

As discussed previously, telomerase inhibitors could prove a possible venue in treatment of diseases that are inherently dependent on cell division and mitosis like cancer. Phase II trials (Baerlocher et al., 2015; Baerlocher et al., 2019; Mascarenhas et al., 2021), and a phase III trial that led to imetelstat’s approval against low-to intermediate-1 risk myelodysplastic syndromes with transfusion-dependent anaemia (Platzbecker et al., 2024) were previously conducted. Additionally, several studies investigated the therapeutic effects and adverse events of imetelstat, which are summarized in Table 1. In a 2015 study, Baerlocher et al. as part of a phase II study established reduction in growth of megakaryocyte colony forming units (CFUs) in essential thrombocythemia (ET) following imetelstat treatment regardless of driver mutation at 1 month after starting imetelstat treatment (Baerlocher et al., 2015). In the same phase II study in 2019 (Baerlocher et al., 2019), they established that the dose-dependent inhibitory effect of imetelstat had no significant impact on cytokine-stimulated megakaryocytes of healthy individuals, which they attributed to the possibility of higher hTERT levels in ET subjects (Baerlocher et al., 2019). Another study by Baerlocher et al. focused on side effects of imetelstat, mainly cytopenia resulting from treatment and concluded that toll like receptors (TLRs) are not activated by imetelstat, and the side effects (cytopenia) may occur due to effects on the stem cell pool (Baerlocher et al., 2020), stating a need of further investigation. Mascarenhas et al. confirmed a reduction variant allele frequency in JAK2V617F, CALR, or MPL driver mutations and an increase in survival after administrating imetelstat in myelofibrosis patients (Mascarenhas et al., 2021). The patients were given one of two doses of imetelstat (4.7 mg/kg and 9.4 mg/kg), both doses showed improvements in the form of reduction of spleen size, with the higher dose also showing higher expression of side effects like night sweats, itchiness, pain under left ribs and bone/muscle pain, thus providing insight into imetelstat therapeutic effects but also the possible dose-dependent adverse effects of the treatment (Mascarenhas et al., 2021). Barwe et al. showed the effectiveness of imetelstat in treating acute myeloid leukaemia (AML) alone or combined with azacitidine or chemotherapy, as it enhanced the median survival of mice engrafted with xenografts generated from pediatric patients undergoing treatment for AML (Barwe et al., 2022). The study showed no significant impact of imetelstat treatment on bone marrow cells taken from healthy pediatric subjects (Barwe et al., 2022). Bruedigam et al. also investigated acute myeloid leukaemia (AML) using patient-derived xenograft mouse models (PDX) and found that imetelstat increased lipid peroxidation and increased expression of acyl-CoA synthetase long-chain family member 4 (ACSL4) and fatty acid desaturase 2 (FADS2). ACSL4 and FADS2 loss of function through using single guided RNA (sgRNA) editing, forming imetelstat-resistant variants, concluded that their activity in catalysing polyunsaturated fatty acids (PUFAs) may lead to their accumulation. As PUFAs are susceptible to peroxidation, their accumulation explains the imetelstat-mediated ferroptosis (Bruedigam et al., 2024). Fischer-Mertens et al. discovered positive effects for imetelstat in telomerase positive neuroblastoma, alone or in combination chemotherapy (etoposide or doxorubicin), highlighted by tumour growth reduction *in vivo* and improved mouse survival rates in PDX (Fischer-Mertens et al., 2022). In this study, 6-thio-dG showed a similar survival profile under both conditions (alone and with combination therapy), though when combined with ceritinib, an anaplastic lymphoma kinase (ALK) inhibitor, it showed weaker synergy (Fischer-Mertens et al., 2022). While these studies reinforce imetelstat’s role in cancer treatment, its side effects; cytopenia (Baerlocher et al., 2020), bone/muscle pain, night sweats and rib pain that are dose dependent (Mascarenhas et al., 2021); raise daunting risks, which limits its use to specific cases. Further studies are needed for specific tissue targeting and localization.

**TABLE 1:** Summary of research studies on imetelstat.

Year	Condition	Outcomes/Mechanism	References
2015–2019	Essential thrombocythemia	Observed reduction of growth of megakaryocytes irrespective of driver mutation on treatment (*in vitro*), phase II study	Baerlocher et al. (2015), Baerlocher et al. (2019)
2020	Cytopenia associated with imetelstat treatment (side effect	Toll like receptors (TLRs) is not activated by imetelstat therapy, and cytopenia is suggested to be due to effects on stem cell pool	Baerlocher et al. (2020)
2021	Myelofibrosis	Improvement in survival and reduction in spleen size (phase II study), dose-dependent side effects	Mascarenhas et al. (2021)
2022	Acute myeloid leukaemia	Imetelstat alone or in combination with azacitidine improved median survival rate of mice, and reduced number of leukaemia stem cells	Barwe et al. (2022)
2022	Neuroblastomas	Imetelstat alone or in combination with chemotherapy in telomerase positive neuroblastomas reduced tumour growth *in vitro* and improved survival rate in mouse xenografts	Fischer-Mertens et al. (2022)
2024	Acute myeloid leukaemia	ACSL4 and FADS2 were found to be highly expressed in imetelstat responsive cells. The enzymes catalyze PUFAs which explain peroxidation and imetelstat-mediated ferroptosis	Bruedigam et al. (2024)
2024	Myelodysplastic syndromes	Phase 3 trial for imetelstat approval by FDA. It showed better outcomes for patients with relapse after erythropoiesis treatment, non-respondent patients or those who were ineligible	Platzbecker et al. (2024)

## Telomerase inactivation

**FIGURE 6:**
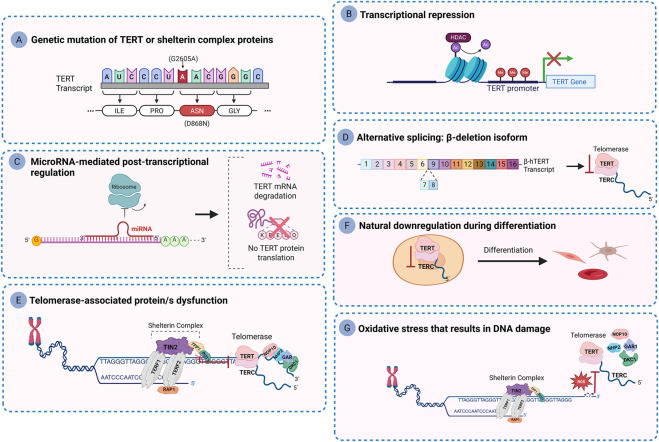
Mechanisms of telomerase Inactivation. **(A)** Genetic mutations: Point mutations such as G2605A lead to an aspartic acid (Asp) to asparagine (Asn) substitution (D868N), impairing telomerase function. **(B)** Transcriptional repression: The hTERT promoter is silenced by histone deacetylases (HDACs) and DNA hypermethylation, leading to reduced telomerase expression. **(C)** MicroRNA-mediated regulation: MicroRNAs such as miR-138 and miR-128 target hTERT mRNA, suppressing translation and lowering telomerase levels. **(D)** Alternative splicing: The *β*-deletion isoform of hTERT produces a truncated, non-functional variant, preventing telomerase activity. **(E)** Dysfunction of telomerase or its associated proteins: Structural or functional defects in telomerase or its accessory proteins, including components of the shelterin complex, disrupt proper telomere maintenance. **(F)** Telomerase downregulation during cellular differentiation: During differentiation, hTERT expression declines, leading to telomerase silencing and limiting telomere elongation in most somatic cells. **(G)** Oxidative stress-induced DNA damage: DNA lesions caused by oxidative stress inhibit telomerase-mediated telomere elongation.

Telomerase inactivation is not exclusive to telomerase-targeted drugs but represents a naturally regulated process essential for maintaining controlled cell proliferation. While telomerase activation supports chromosomal stability by preserving telomere length and capping, loss of telomerase function or telomere uncapping can promote genomic instability and trigger DNA damage response (O’sullivan and Karlseder, 2010; Lorbeer and Hockemeyer, 2020). Mutations in telomerase-related genes, such as TERT, TERC, and dyskerin/DKC1, can impair enzymatic activity and are linked to DC (Figure 6A) (Wong and Collins, 2006; Du et al., 2009). A major regulatory mechanism involves repression of TERT gene transcription through promoter hypermethylation and histone deacetylation (Figure 6B) (Dogan and Forsyth, 2021; Leão et al., 2018). However, in cancer cells, methylation of the TERT promoter paradoxically activates hTERT expression by altering chromatin accessibility, thereby enhancing telomerase activity without necessarily altering telomere length (Lee et al., 2020). Beyond epigenetics, transcription factors such as c-Myc, Sp1, and Mad/Max complexes also regulate TERT expression by binding to the promoter region and either activating or repressing transcription (Dogan and Forsyth, 2021; Leão et al., 2018; Kyo et al., 2000; Xu et al., 2001; Zhao et al., 2014).

Post-transcriptional regulation provides an additional layer of control. MicroRNAs such as miR-138 and miR-128 suppress TERT by binding to its 3′-UTR, promoting mRNA degradation or inhibiting translation (Figure 6C) (Leão et al., 2018; Agarwal et al., 2015). Moreover, alternative splicing of TERT generates multiple isoforms with distinct functions and catalytic capacities. For instance, deletion of exons 7–8 yields the *β*-isoform, a catalytically inactive transcript that acts in a dominant-negative fashion to reduce overall telomerase activity (Figure 6D) (Slusher et al., 2020; Plyasova and Zhdanov, 2021).

In addition to TERT regulation, telomere-associated proteins such as TPP1 play a crucial role in telomerase recruitment and processivity (Figure 6E) (Sekne et al., 2022; Grill et al., 2018; Waitkus et al., 2024). Dysfunction or loss of TPP1 and other shelterin components (e.g., TIN2, POT1) impairs telomere maintenance, not by abolishing telomerase catalysis directly, but by hindering its recruitment to telomeric ends (Wang et al., 2007; Abreu et al., 2010; Nandakumar et al., 2012).

Telomerase downregulation also occurs under physiological conditions. During cellular differentiation, human somatic cells naturally silence telomerase to restrict proliferative potential and enforce replicative limits (Figure 6F) (Newbold, 2002). Interestingly, cellular stress can modulate telomerase-mediated telomere elongation. Specifically, oxidative stress increases the oxidized nucleotide pool, including 8-oxo-7,8-dihydro-2′-deoxyguanosine triphosphate (8-oxo-dGTP). Incorporation of 8-oxo-dGTP by telomerase into telomeric DNA produces 8-oxo-dG lesions that act as a chain terminator, thereby inhibiting telomere elongation without abolishing telomerase activity. Conversely, pre-existing 8-oxo-dG lesions can destabilize telomeric G-quadruplex structures, paradoxically enhancing telomerase access and promoting elongation under certain oxidative conditions (Figure 6G) (Fouquerel et al., 2016).

As cells age, telomere shortening caused by the end-replication problem and oxidative stress, together with the absence of telomerase activity, leads to senescence in most human somatic cells (Leão et al., 2018). Collectively, these mechanisms demonstrate the intricate, multi-layered regulation of telomerase, highlighting its dual role in preventing oncogenic transformation while safeguarding genomic integrity and cellular homeostasis.

## Conclusion and remarks

Progress in structural biology technologies and techniques have enabled the detection of large and dynamic complexes in high eukaryotic systems, such as telomerase in humans. This progress is evident when examining the recent discoveries in telomerase structure, which has evolved from solving the structure of peptides to major parts of the holoenzyme, allowing for in-depth characterization of telomerase. It also advanced our understanding of the interactions that regulate the holoenzyme’s function or cessation. These interactions mediate the role of telomerase in aging and diseases. To address these issues, research is advancing to solve the telomerase structure and develop novel strategies for telomerase regulation.

Despite the significant progress in resolving the structure of the human telomerase holoenzyme, many interactions remain unresolved. For instance, POT1 domains (OB3 and HJRL) and the CTE of TPP1, which interact with it, are still not fully characterized (Sekne et al., 2022). Additionally, many regions in the structure of GAR1’s CTE remain unresolved, as well as the CTE domain of dyskerin (Ghanim et al., 2021; Ghanim et al., 2024; Liu B. et al., 2022; Wan et al., 2021). Also, it becomes evident that hTERT itself has missing loops, particularly a large disordered or unstructured loop spanning around 140 amino acids (TEN: 180–200, PAL region: 201–321 aa) (Ghanim et al., 2021; Liu B. et al., 2022; Sekne et al., 2022; Wan et al., 2021). Furthermore, no studies have successfully solved an intact and high-resolution structural model that has all the components of the catalytic and H/ACA lobes together. This is attributed to the highly complex dynamics of the telomerase holoenzyme, the high flexibility some regions such as the PAL region, and telomerase low abundance (Ghanim et al., 2024).

These limitations also extend to the development of human telomerase-targeting drugs. This is clearly illustrated by imetelstat, which has only recently become the first FDA-approved telomerase inhibitor, and its approval is limited to use in low-to intermediate-1 risk myelodysplastic syndrome (Platzbecker et al., 2024). Additionally, studies on its side effects are still ongoing. Some findings suggest potential benefits for diseases, such as neuroblastoma, while others highlight adverse effects, like thrombocytopenia, that even led to premature study closure (Fischer-Mertens et al., 2022; Salloum et al., 2016). Yet, many of these effects remain poorly understood, and the reasons behind their emergence are not well defined. These uncertainties limit the broader application of imetelstat in a therapeutic capacity. Overall, there is a need for improved purification and modelling strategies to fully resolve the telomerase structure to understand its biogenesis, function and regulation. Further research on imetelstat and telomerase-targeted drugs is also needed to comprehensively characterize their mechanisms of action and potential side effects.
